# Biologics and JAK inhibitors for atopic manifestations in inborn errors of immunity: a systematic review

**DOI:** 10.3389/falgy.2026.1867183

**Published:** 2026-10-07

**Authors:** Mahnaz Jamee, Roba Maher Al-Gaweesh, Amirhossein Hajialigol, Sally Gouda Mohammed, Peyman Tirandazi, Abdullah A. Alangari, Rasha El-Owaidy

**Affiliations:** 1 Human Immune Disorders Laboratory, Garvan Institute of Medical Research, Sydney, NSW, Australia; 2 St Vincent’s Clinical Campus, School of Clinical Medicine, Faculty of Medicine and Health, UNSW Sydney, Sydney, NSW, Australia; 3 Pediatric Allergy, Immunology and Rheumatology Unit, Children’s Hospital, Ain-Shams University, Cairo, Egypt; 4 Student Research Committee, Alborz University of Medical Sciences, Karaj, Iran; 5 Department of Computer Science, Yazd University, Yazd, Iran; 6 Department of Pediatrics, College of Medicine, King Saud University, Riyadh, Saudi Arabia

**Keywords:** atopy, biologics, Inborn error of immunity, JAK inhibitor, monoclonal antibody

## Abstract

**Background:**

Atopy might be the initial or dominant manifestation in patients with inborn errors of immunity (IEIs). Biologics and Janus kinase (JAK) inhibitors have emerged as targeted options for atopic disorders in IEIs refractory to conventional treatment.

**Methods:**

We conducted a systematic review of PubMed, Web of Science, and Scopus for human studies reporting biologics or JAK inhibitors used specifically to treat atopic manifestations in IEIs. Data on genotypes, clinical phenotypes, treatments, outcomes, adverse events, and laboratory changes were extracted and synthesized. The databases were last searched in April 2026. Study quality was appraised with the Joanna Briggs Institute checklists and the certainty of evidence was rated following GRADE principles.

**Results:**

Eighty-one studies comprising 172 patients were included. The most common IEIs were STAT3 loss-of-function (LOF) disease (*n* = 52, 30.2%), Netherton syndrome (*n* = 32, 18.6%), and DOCK8 deficiency (*n* = 19, 11.0%). Dupilumab (*n* = 120, 69.8%) was the most frequently reported agent, followed by JAK inhibitors (*n* = 21, 12.2%) and omalizumab (*n* = 16, 9.3%). A complete or major clinical response was described in most published patients treated with dupilumab, together with improvement in skin disease in some patients. Omalizumab was also associated with a favorable outcome in atopic dermatitis and asthma, with steroid-sparing effects and few reported adverse events. Responses to JAK inhibitors were most often described in patients with JAK-STAT pathway defects, although adverse events such as anemia were reported in some cases. All proportions are descriptive frequencies among published patients with available outcome information and are not estimates of treatment effectiveness.

**Conclusion:**

Published case reports and small series suggest that biologics and JAK inhibitors may provide clinically meaningful control of selected atopic or atopic-like manifestations in some patients with IEIs. Dupilumab has the largest published experience, while JAK inhibitors may be particularly relevant when the underlying disorder involves JAK-STAT pathway activation. Due to the lack of evidence, treatment should remain individualized, genotype-informed, and accompanied by careful infection surveillance and longitudinal outcome assessment.

## 1 Introduction

Inborn errors of immunity (IEIs) are a group of 559 inherited disorders, involving 508 genes and 17 phenocopies, with defects in the structure or function of the immune system (1). While IEIs were classically known to manifest with recurrent infections, there is increasing recognition that immune dysregulation, including autoimmunity, autoinflammation, and atopy, constitutes a major component of their phenotype and may occasionally present in isolation (2, 3). Among IEIs, the category of combined immunodeficiencies (CIDs), particularly syndromic forms, consistently manifests the highest burden of atopic disorders (4–6). The mechanisms driving these manifestations and the implicated genes are summarized elsewhere (7). In brief, atopic manifestations in IEIs arise through several partly overlapping mechanisms: excessive or unopposed type 2 signaling (e.g., STAT6 and IL4R gain-of-function), constitutive activation of the JAK-STAT pathway (e.g., JAK1 and STAT5B gain-of-function), impaired regulatory T-cell development or function (e.g., FOXP3, CTLA4, and IL2RA defects), defective T-cell receptor and NF-kB signaling in CIDs with severe atopy (e.g., DOCK8, CARD11, CARMIL2, ARPC1B, and WAS deficiency), impaired STAT3-dependent signaling in the hyper-IgE syndromes (e.g., STAT3 loss-of-function, ZNF341, IL6ST, IL6R, and PGM3 defects) and epithelial-barrier failure with secondary type 2 sensitization (e.g., SPINK5, FLG, DSG1, DSP, and CDSN defects) (8).

The overall prevalence of atopic disorders in IEIs varies across literature. A systematic scoping review of 3,050 IEI patients, derived from studies reporting allergic or atopic features, estimated an overall atopic prevalence of 26.9% and a confirmed allergy rate of 7.4% (4). Another survey on 8,450 IEI patients from 23 countries showed that a median of 16.3% of patients experienced allergic manifestations during the course of their disease, with 8% having allergic symptoms as their initial presentation (9). These findings are consistent with a cohort study of 313 patients with IEI, in which 16.3% exhibited atopic manifestations, nearly 40% of whom presented with atopy as the initial manifestation (10). When atopy occurs as an initial or isolated feature, differentiating monogenic from polygenic causes is challenging, highlighting the importance of awareness aiming at early identification and proper intervention.

Atopy in IEIs is often considered part of a broader immune dysregulation phenotype with distinct genetic and signaling pathways, which affects how the immune system responds to therapies (11). Therefore, the standard immunomodulatory treatment developed for atopic disorders in the general population might be inadequate or require adaptation based on the disease pathomechanism. The conventional treatment for atopic dermatitis includes moisturizers, but also immunosuppressants, including steroids and calcineurin inhibitors, which might raise concerns about safety in immunodeficient patients who suffer from barrier defects and severe skin diseases (12, 13). Targeted therapies offer greater specificity, and a different toxicity profile, than systemic corticosteroids and calcineurin inhibitors. Agents used for atopic disease include dupilumab (IL-4R*α*), omalizumab (free IgE), mepolizumab and benralizumab (IL-5 and IL-5R*α*), tralokinumab (IL-13), tezepelumab (TSLP), and the JAK inhibitors such as ruxolitinib, baricitinib, tofacitinib, abrocitinib, and upadacitinib. This is especially relevant in IEI, which often involves defined defects in specific immune pathways and may therefore be amenable to pathway-directed therapy (13, 14). They may also serve as an adjunct to immunoglobulin replacement or as a bridge therapy for patients with IEIs who are later candidates for hematopoietic stem cell transplantation (HSCT), which has been shown to reduce atopic manifestations in some of these disorders (14).

The use of biologics and JAK inhibitors to control atopic manifestations in IEIs remains incompletely characterized. Current evidence is largely limited to case reports, case series, and heterogeneous cohorts, with no comprehensive synthesis available. Therefore, this systematic review was designed to identify which agents have been used and in which genetic disorders, assess the reported effects on clinical and laboratory outcomes, and evaluate the reported adverse events and safety profiles.

## 2 methods

### 2.1 study design, search strategy, and data sources

We performed a comprehensive literature search using three databases: PubMed, Web of Science, and Scopus. A combination of keywords was applied for a systematic search. The databases were searched from inception to April 2026. Complete database-specific strategies and the number of records retrieved from each database are provided in Supplementary Table S1. The review is reported in accordance with the PRISMA 2020 statement. The review was not registered prospectively; it followed a written protocol agreed among the authors before screening began, and no eligibility criterion or outcome was altered after data extraction started. The keywords were selected as different terminologies for targeted therapy, atopy or allergy, and inborn errors of immunity, including genes associated with atopic manifestations in the latest IUIS classification. Also, keywords related to hereditary or acquired angioedema were excluded because this large group of patients presents in a distinct way that might distort global analysis. Patients with cystic fibrosis were excluded irrespective of the agent or indication, as the use of omalizumab for allergic bronchopulmonary aspergillosis (ABPA) in this population has also recently been evaluated in a comprehensive meta-analysis (15). The keywords were as follows:

“Janus kinase inhibitors”, “monoclonal antibodies”, “Ruxolitinib”, “Tofacitinib”, “Upadacitinib”, “Abrocitinib”, “Tezepelumab”, “Omalizumab”, “Dupilumab”, “Mepolizumab”, “Benralizumab”, “Reslizumab”, “primary immunodeficiency diseases”, “inborn error of immunity”, “CARD11”, “MALT1”, “WAS”, “WIPF1”, “ARPC1B”, “DOCK8”, “CARMIL2”, “NCKAP1L”, “STK4”, “MSN”, “JAK1”, “STAT1”, “STAT3”, “STAT5B”, “STAT6”, “IL2RA”, “IL4RA”, “TGFBR1”, “TGFBR2”, “ERBB2IP”, “IL6ST”, “IL6R”, “ZNF341”, “FOXP3”, “TBX21”, “RAG1”, “RAG2”, “DCLRE1C”, “ADA”, “IL2RA”, “IL7RA”, “CHD7”, “LIG4”, “ZAP70”, “22q11deletion syndrome”, “IL2RG”, “TBX1”, “IKZF1”, “CARD14”, “CARMIL2”, “RFXANK”, “PGM3”, “FLG”, “CDSN”, “DSG1”, “DSP”, “SPINK5”, “PLCG2”, “ADGRE2”, “TPSAB1”, “KIT”, “hypersensitivity”, “Allergy”, “allergic reaction”, “atopy”, “atopic disorder”, “atopic dermatitis”, “food allergy”, “eczema”, “urticaria”, “allergic asthma”, “eosinophilic pneumonia”, “eosinophilic esophagitis”, “Ichthyosis”, “allergic rhinitis”, and “chronic rhinosinusitis”. Moreover, we reviewed the reference list of full-text articles and major reviews and manually added additional studies.

### 2.2 Study selection

During the first round of screening, the titles and abstracts were screened independently in duplicate with disagreements resolved by discussion and, where necessary and were evaluated to find studies on patients with inborn errors of immunity in which any types of biologics or JAK inhibitors were indicated. In the second round of screening, the full text of included studies was examined to precisely determine the eligibility based on the inclusion and exclusion criteria. The inclusion criteria were English language, human studies, report of at least one patient with genetic diagnosis of IEI receiving biologics and/or JAK inhibitors specifically for the atopic manifestations. For the purpose of this review, an inborn error of immunity was defined as a monogenic disorder listed in the 2024 update of the IUIS Expert Committee classification (1) together with monogenic disorders subsequently reported as causing immune dysregulation with a defined immunological phenotype (Supplementary Table S2). Every included disorder was mapped to that classification, and disorders were grouped as: 1. IUIS-listed IEIs, 2. primary atopic disorders and epithelial-barrier defects, including Netherton syndrome, and 3. syndromic or incompletely characterized conditions. Conditions not meeting this definition were excluded. Eligibility was restricted to patients with a sequencing-confirmed molecular diagnosis, except for three patients with a clinically definitive hyper-IgE phenotype in whom sequencing identified only a variant of uncertain significance; these are identified separately and are excluded from the sensitivity analysis restricted to genetically confirmed IUIS-listed disorders (Supplementary Table S3). Basic and translational articles conducted only on animal models or *in vitro*, conference abstracts, commentaries, reviews, chapter books, studies in languages other than English, and indications other than atopic disorders for the prescription of biologics and JAK inhibitors were excluded. The corresponding authors of potential studies and included studies were contacted for missing data.

### 2.3 Data extraction and analysis

An Excel spreadsheet was designed, and data extraction was distributed among four researchers using a piloted standardized form, with cross-checking of extracted records against the source reports by a second researcher and resolution of discrepancies by consensus. This was performed on the “included” articles. Data related to publication year, number of participants, patients’ genetic, demographic, clinical, and immunological data, and treatment details, such as the type of targeted therapy, dosage, duration, side effects, and outcomes, were extracted from each article. Patients reported in more than one publication were identified and counted only once. Potential duplicates were sought among reports sharing gene and variant, sex, age at treatment initiation, country and treating center, author group, treatment agent, and distinctive clinical features. Where overlap was suspected but could not be confirmed, the patient was counted once and the more complete report retained; where completeness was equivalent, the most recent report was retained; overlapping publications and the retained source for each patient are listed in Supplementary Table S4. Patients exposed sequentially to more than one agent were counted once in the overall cohort but appear in the denominator of each agent-specific analysis. In addition to demographic, genetic, clinical, and treatment variables, concomitant systemic immunosuppression, immunoglobulin replacement, antimicrobial prophylaxis, topical treatment, nutritional support, prior targeted therapy, and the timing of treatment relative to HSCT were extracted, so that responses occurring during monotherapy could be distinguished from those occurring during combination therapy. A sensitivity analysis restricted to monotherapy was prespecified but could not be computed, because the source reports rarely give enough temporal detail to define an assessment window; this is acknowledged as a limitation.

Central and descriptive statistics were reported for quantitative data. Median and interquartile ranges (IQR) were reported for variables with abnormal distribution. For variables with a normal distribution pattern, mean ± Standard Deviation (SD) was used for the data report. All statistical analyses were performed using the SPSS software (v. 26.0, Chicago, IL). Figures were generated using the BioRender and SankeyMATIC websites.

### 2.4 Outcome definitions

Clinical response was classified into four categories, applied to the descriptions given in the source publications: a complete or major clinical response, defined as near-complete resolution or marked improvement of the treated manifestation sustained to the last reported follow-up; a partial response, defined as documented improvement insufficient to meet that threshold; a response followed by relapse, defined as initial improvement with subsequent recurrence between doses, on dose spacing, or after discontinuation; and no reported response. These categories were defined post hoc, before the outcome data were analyzed, and were applied to the descriptions given in the source publications by two reviewers independently, with disagreements resolved by discussion.

Outcomes were recorded separately for each atopic manifestation rather than as a single global outcome, and were stratified according to whether they were supported by an objective or validated measure, such as EASI, SCORAD, Investigator Global Assessment, affected body surface area, pruritus scores, quality-of-life instruments, spirometry, exacerbation or systemic corticosteroid counts, supervised oral food challenge, or endoscopic or histological findings, or by author-reported global improvement alone. Atopic manifestations were further classified by level of diagnostic confirmation, distinguishing clinically diagnosed atopic dermatitis from eczematous dermatitis without validated criteria, challenge-confirmed or convincingly immediate IgE-mediated food allergy from sensitization alone, objectively confirmed asthma from clinically described asthma, and treating eosinophilic gastrointestinal inflammation and non-specific hyper-IgE or eosinophilia as separate categories. An elevated total IgE or a positive specific IgE was not taken to establish clinically relevant allergy.

### 2.5 Risk of bias and certainty of evidence

The methodological quality of the included studies was appraised with the Joanna Briggs Institute critical appraisal checklists for case reports and for case series, and with the Joanna Briggs Institute critical appraisal checklist for cohort studies for retrospective cohorts (16). Appraisal covered the distribution of study designs, completeness of outcome ascertainment, duration of follow-up, clarity of the atopic diagnosis, certainty of the genetic diagnosis, the quality of adverse-event reporting, and whether treatment response was measured objectively. Appraisal was performed independently by three reviewers, with disagreements resolved by discussion; item-level judgements are given in Supplementary Tables S5a, b. Because all included reports were non-comparative and most described single patients, the certainty of the evidence for all outcomes was rated as very low following GRADE principles, with further downgrading for risk of bias, indirectness, imprecision, and a high likelihood of publication bias. The frequencies reported below are therefore proportions of published patients with available outcome information and are not estimates of treatment effectiveness in the underlying population.

## 3 Results

### 3.1 Study characteristics

The primary systematic search identified 5,222 articles, of which 997 were excluded due to duplication and other reasons (Figure 1). The titles and abstracts of the remaining 4,225 articles were screened, and 4,096 were deemed irrelevant. Full-text assessment was conducted for 129 articles based on the inclusion criteria, resulting in the inclusion of 71 studies. An additional 10 articles were identified through screening of major review articles and other resources. In total, 81 articles were included in the qualitative and quantitative analyses for Dupilumab (17–66), Omalizumab (67–77), JAK inhibitors (78–89), and other agents [Benralizumab (90), Infliximab (91, 92), Secukinumab (93–95), Tralokinumab (96), and Ustekinumab (97)].

**Figure 1:**
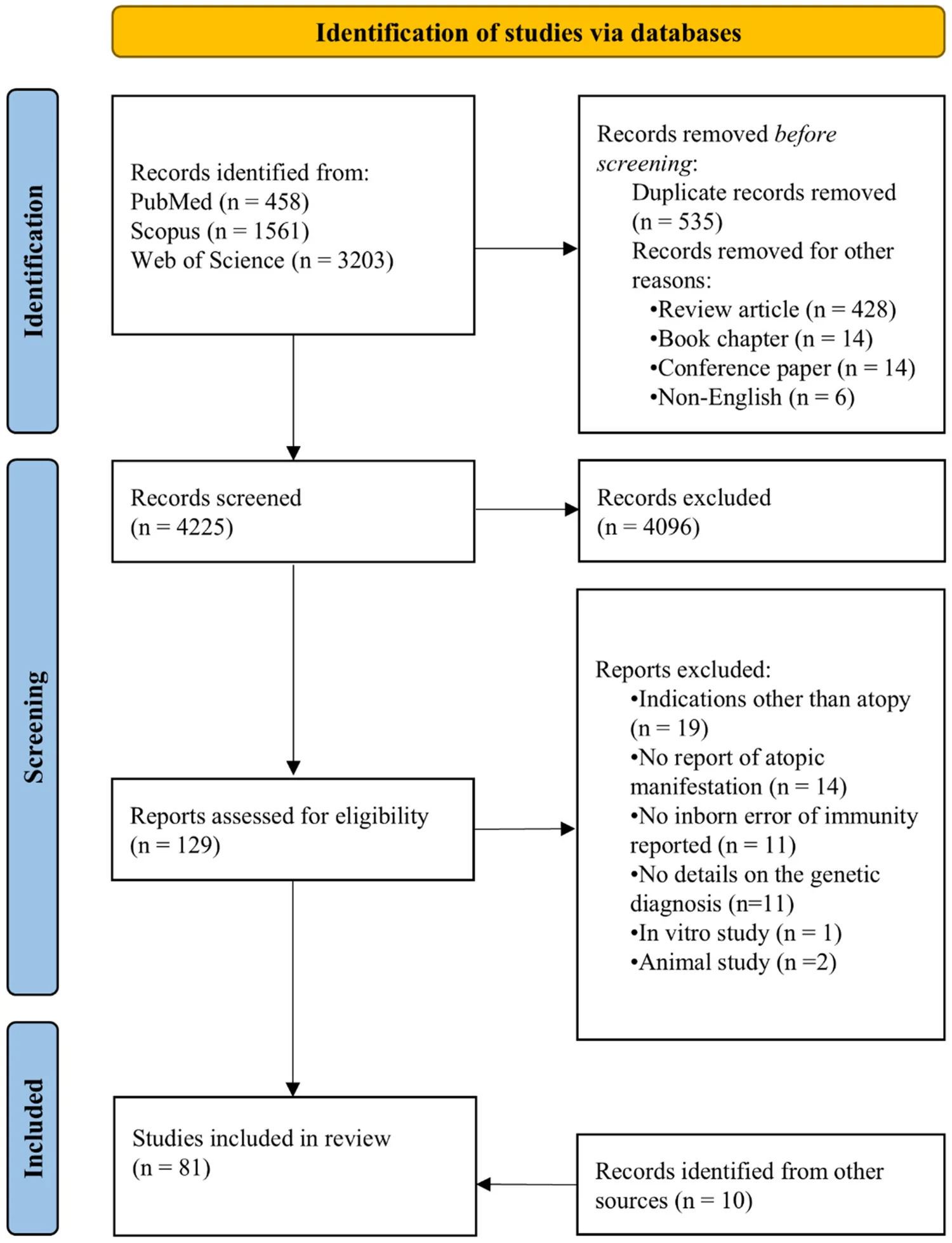
The PRISMA flowchart for identification, screening and inclusion of studies.

### 3.2 Targeted immunomodulators for atopic manifestations in inborn errors of immunity

#### 3.2.1 General

A total of 172 patients were included. Age was reported for 157 patients and ranged from 2 months to 58 years. Sex was reported for 140 patients, of whom 76 (54.3%) were male and 64 (45.7%) female. Most reports were from the United States (*n* = 40, 23.3%), France (*n* = 39, 22.7%), China (*n* = 14, 8.1%), and Saudi Arabia (*n* = 12, 7.0%). At treatment initiation, 101 of 157 patients (64.3%) with a reported age were children, with a median (IQR) age of 10 (6-14) years, and 56 (35.7%) were adults, with a median (IQR) age of 27.6 (21.0-33.3) years. Consanguinity was reported for only 22 patients, of whom 9 (40.9%) had consanguineous parents.

Disorders were grouped as IUIS-listed inborn errors of immunity (*n* = 133), epithelial-barrier disorders (*n* = 32, all Netherton syndrome) and syndromic or incompletely characterized conditions (*n* = 7). The most common diagnoses were STAT3 loss-of-function (LOF) disease (*n* = 52, 30.2%), Netherton syndrome (*n* = 32, 18.6%), and DOCK8 deficiency (*n* = 19, 11.0%), followed by JAK1 gain-of-function disease (*n* = 15, 8.7%), CARD11-associated atopy with dominant interference of NF-*κ*B signaling (CADINS) (*n* = 13, 7.6%), and other disorders. Multiple atopic manifestations were recorded in 83 patients (48.3%). Among the 88 patients with a single recorded manifestation, 77 (87.5%) had atopic dermatitis or eczematous dermatitis. In patients with multiple atopic manifestations, the most common overlap was between atopic dermatitis and asthma in 47 (56.6%) and between atopic dermatitis and food allergy in 36 (43.4%) patients. The most frequently reported agents were dupilumab (*n* = 120, 69.8%), JAK inhibitors (*n* = 21, 12.2%), and omalizumab (*n* = 16, 9.3%), which are described in more detail in Sections 3.2.2–3.2.4, respectively. Other agents were secukinumab (*n* = 7, 4.1%) and infliximab (*n* = 3, 1.7%), both given in Netherton syndrome for the broader inflammatory and ichthyosiform skin phenotype rather than for a discrete atopic manifestation, and, each in a single patient (0.6%), tralokinumab and benralizumab for atopic dermatitis and asthma in STAT3 LOF disease, ustekinumab for the skin phenotype of Netherton syndrome, mepolizumab for asthma and reslizumab for eosinophilic esophagitis, both in STAT3 LOF disease. The mechanism of action of these agents is illustrated in Figure 2.

**Figure 2:**
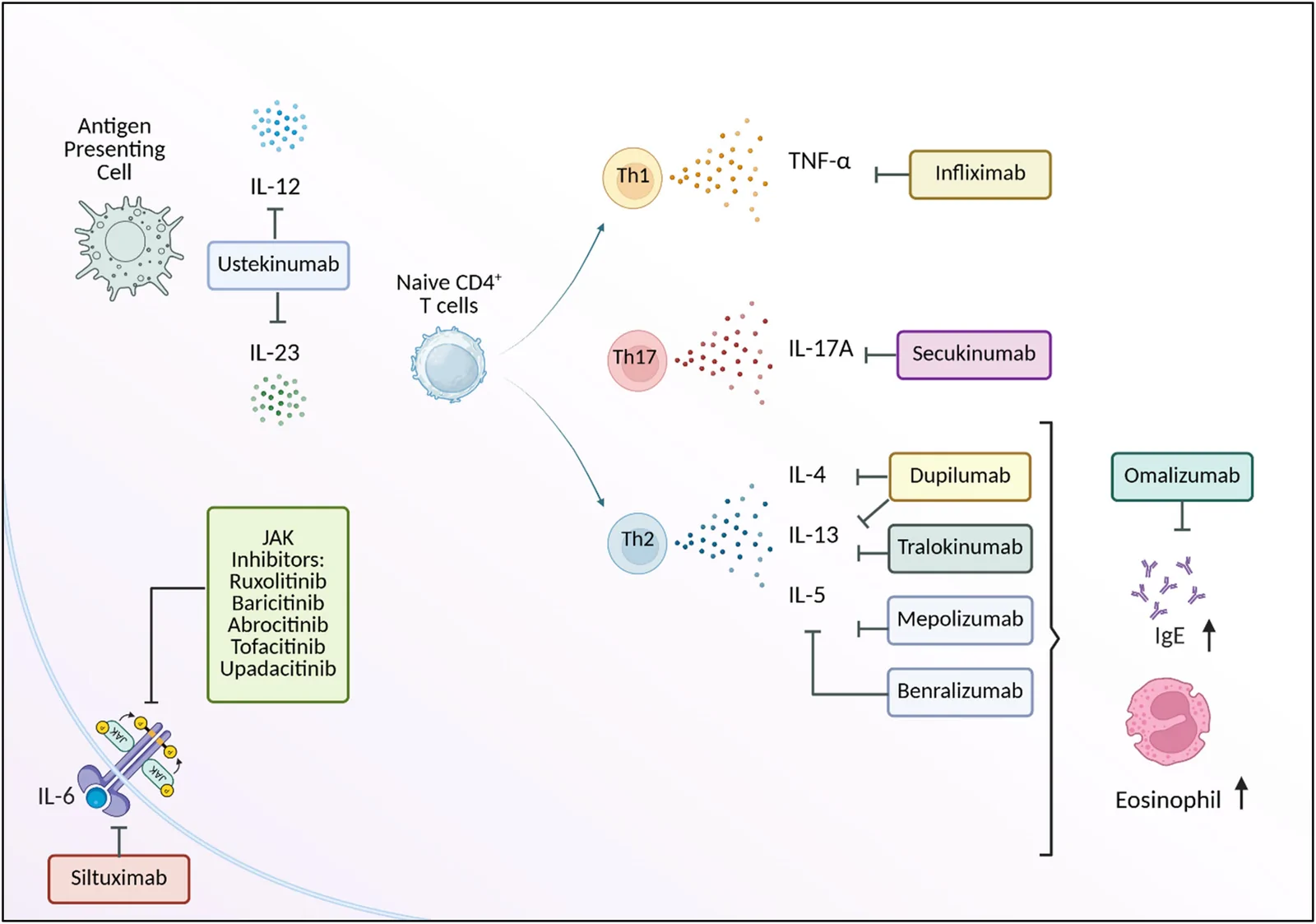
Mechanisms of action in selected biologics and JAK inhibitors used for atopic manifestations in inborn errors of immunity.

The duration of post-administration follow-up was reported for 160 patients, with a median (IQR) of 12 (6-21) months. Paired pre- and post-treatment measurements were available for IgE in 60 and for absolute eosinophil count in 39 patients. A reduction was recorded in 48 of 60 (80.0%) and 25 of 39 (64.1%), respectively. Absolute values are reported in the extraction dataset where the source provided them; a reduction was distinguished from normalization only where the source stated the reference range. A reduction in IgE or eosinophil count is a biological response marker of uncertain clinical significance and has not been validated as a surrogate for clinical benefit in IEIs. At treatment initiation, 23 patients (13.4%) had a documented history of previous or concurrent treatment with another biologic or JAK inhibitor.

#### 3.2.2 Dupilumab

Dupilumab is a human IgG4 monoclonal antibody that targets the shared alpha subunit of IL-4 and IL-13 receptor complexes, blocking type 2 inflammation (98). Type 2 inflammation is the central mechanism involved in allergy. Its initiation and maintenance are mostly carried out by type 2 helper T lymphocytes and cytotoxic T cells from the adaptive immune system, and also mast cells and type 2 innate lymphoid cells from the innate immune system. These cells then produce cytokines such as IL-4, IL-5, IL-13, and IL-31 to recruit eosinophils and basophils and enhance class switching of B cells to the IgE-producing plasma cells (99).

We found that dupilumab was prescribed for 120 patients, most commonly for STAT3 LOF disease (*n* = 42, 35.0%), DOCK8 deficiency (*n* = 18, 15.0%), Netherton syndrome (*n* = 17, 14.2%), and CARD11-associated atopy with dominant interference of NF-*κ*B signaling (CADINS) (*n* = 12, 10.0%). Atopic dermatitis or eczematous dermatitis (*n* = 116, 96.7%), asthma (*n* = 35, 29.2%), and food allergy (*n* = 26, 21.7%) were the most common indications. Sixty patients (50.0%) had multiple atopic manifestations. Dupilumab was used as a bridge to HSCT in 7 patients (5.8%), including 5 patients with DOCK8 deficiency, one patient with STAT3 LOF mutation, and another patient with hyper IgE syndrome and no established genetic diagnosis. The median (IQR) age at treatment initiation was 13 (8-22) years, with a minimum and maximum of 2 months and 52 years, respectively. The duration of post-administration follow-up was reported for 113 patients, with a median (IQR) of 12 (6-18) months and a maximum of 6 years.

Treatment outcome was reported in 118 of 120 patients (98.3%) (Figure 3). A complete or major clinical response was described in 97 of 118 published patients with available outcome information (82.2%), a partial response in 11 (9.3%), a response followed by relapse in 6 (5.1%), occurring between injections or after discontinuation, and no reported response in 4 (3.4%). These are descriptive frequencies among published patients, in which favorable outcomes are far more likely to be published than treatment failures; they are not estimates of response rates in the underlying IEI population and should not be compared across agents. Twenty-seven patients (22.5%) did not require continued use of corticosteroids. Nine patients (7.5%) had previous or simultaneous exposure to another biologic or JAK inhibitor, most often omalizumab. In these patients the assessed response occurred during previous or simultaneous exposure to another agent and cannot be attributed to dupilumab alone.

**Figure 3:**
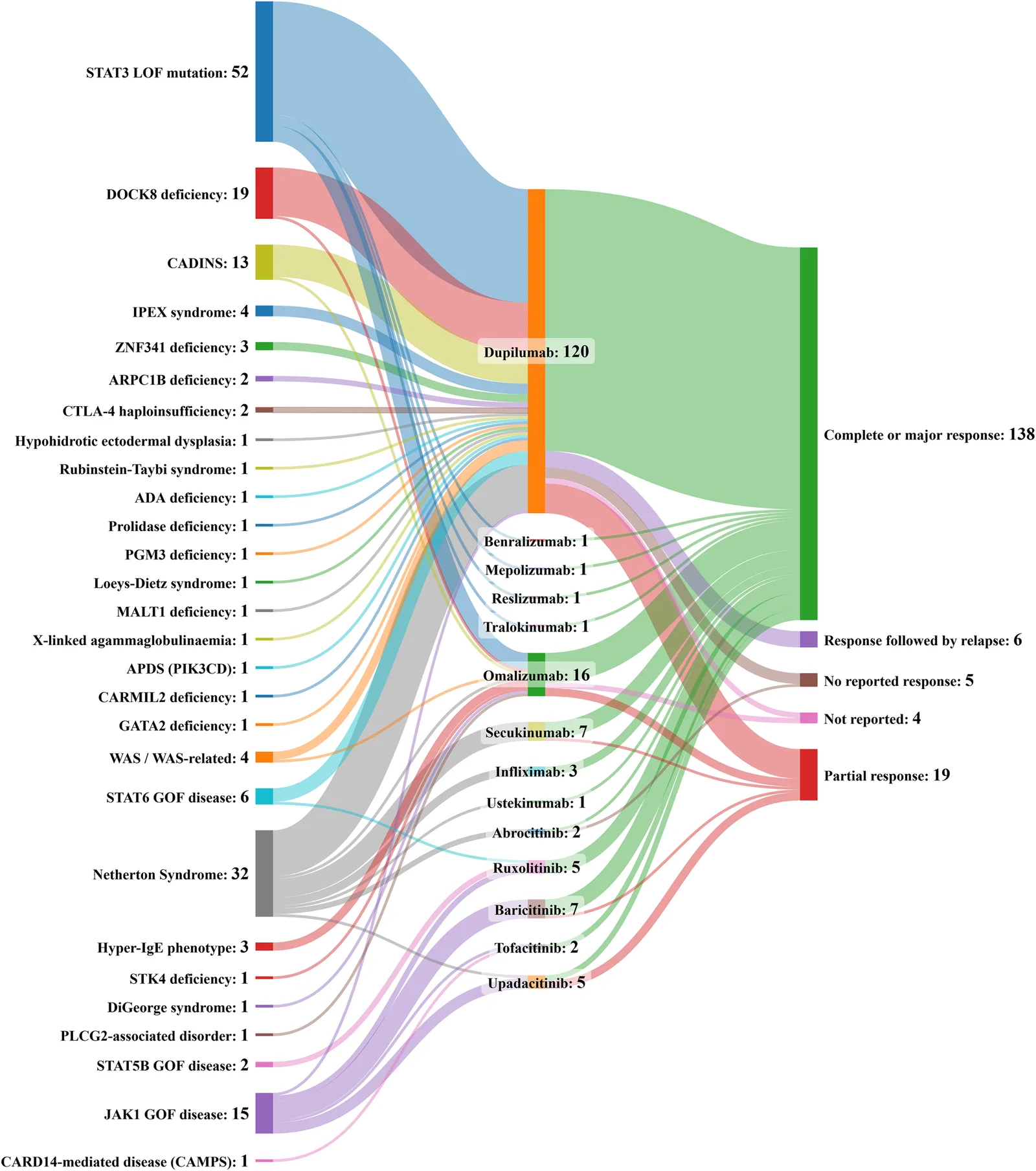
Sankey diagram showing the distribution of biologic and JAK inhibitor use among inborn errors of immunity and their outcome. HIES; hyper IgE syndrome, CVID; common variable immunodeficiency, IPEX; Immune dysregulation, Polyendocrinopathy, Enteropathy, X-linked, Other defects include: Hypohidrotic ectodermal dysplasia, Rubinstein-Taybi Syndrome, ADA deficiency, Prolidase deficiency, IL-6ST defect, ARPC1B deficiency, STAT-3 GOF mutation, Loeys-Dietz syndrome, CTLA-4 haploinsufficiency, PGM3 deficiency, Specific antibody deficiency, ZNF341 deficiency, and CARD11 deficiency, each in one patient. The width of each flow reflects only the number of published patients and does not imply equivalence between diagnoses, outcomes, or the quality of the underlying evidence.

Additional reported clinical benefits were described in 82 patients (68.3%) and mostly included reduced cutaneous infections or normalization of skin microbiome (*n* = 16, 35.5%), weight and/or height gain (*n* = 11, 24.4%), hair and/or nail growth (*n* = 11, 24.4%), decreased systemic infections (*n* = 8, 17.8%), improved functional ability and quality of life (*n* = 7, 15.6%), and improved sleep and daytime concentration (*n* = 4, 8%). Paired pre- and post-treatment measurements were available for IgE in 40 patients and for eosinophils in 27; a reduction was recorded in 34 of 40 (85.0%) and 17 of 27 (63.0%), respectively. Adverse effects were reported in 35 patients (29.2%) and most often consisted of conjunctivitis and transient hypereosinophilia. Adverse-event assessment was explicitly documented in only a minority of reports; absence of reported events in the remainder is not evidence that no events occurred. Reported reductions in infection should also be interpreted cautiously, because improved skin-barrier control, antimicrobial prophylaxis, immunoglobulin replacement, changes in concomitant immunosuppression, and regression to the mean may all contribute.

#### 3.2.3 Omalizumab

Omalizumab is a humanized anti-IgE monoclonal antibody that binds circulating free IgE at the C*ε*3 domain, thereby preventing IgE from engaging Fc*ε*RI on mast cells, basophils, and other effector cells and leading to downregulation of Fc*ε*RI expression and attenuation of allergic inflammation (100).

In this review, omalizumab was used in 16 patients with IEIs, most frequently in those with STAT3 LOF disease (*n* = 6, 37.5%) and in patients with a hyper-IgE phenotype without an established molecular diagnosis (*n* = 3, 18.8%). Most patients (*n* = 10, 62.5%) presented with a single atopic manifestation, usually atopic dermatitis. In total, atopic dermatitis or eczematous dermatitis (*n* = 13, 81.2%), asthma (*n* = 5, 31.2%), food allergy (*n* = 2, 12.5%) and ABPA (*n* = 2, 12.5%) were the most common indications. The median (IQR) age at treatment initiation was 20 (13–32) years, ranging from 5 to 42 years. Post-treatment follow-up duration was available for 13 patients, with a median (IQR) of 9 (4–12) months and a maximum of 9 years.

Treatment outcomes were reported for 14 of 16 patients. A complete or major clinical response was described in 11 of 14 published patients with available outcome information (78.6%) and a partial response in 3 (21.4%). Six patients (37.5%) were able to discontinue corticosteroids. Additional clinical benefits were noted in six patients (37.5%), most often a reduction in the infection rate. Paired pre- and post-treatment IgE measurements were available for 10 patients, of whom 7 were reported to show a reduction. This requires cautious interpretation, because omalizumab forms circulating drug-IgE complexes that are cleared slowly, so measured total IgE typically rises after initiation while free IgE falls, and conventional assays do not distinguish the two fractions. The source reports do not state which fraction was measured, so this figure should be read as descriptive only. An adverse event was reported in one patient. Adverse-event assessment was explicitly documented in only a minority of reports, and the absence of reported events is not evidence that none occurred.

#### 3.2.4 Jak inhibitors

JAK inhibitors block the Janus kinase–signal transducer and activator of transcription (JAK-STAT) pathway, which is the main module used by cytokines to transmit membrane-to-nucleus inflammatory signals. By inhibiting one or more JAK isoforms, JAK inhibitors reduce downstream STAT activation and suppress cytokine-driven immune responses (101).

JAK inhibitors were used in 21 patients, namely baricitinib (*n* = 7, 33.3%), ruxolitinib (*n* = 5, 23.8%), upadacitinib (*n* = 5, 23.8%), and abrocitinib and tofacitinib (each *n* = 2, 9.5%). Ruxolitinib was administered to patients with JAK1 GOF disease (*n* = 2), STAT5B GOF disease (*n* = 2), and STAT6 GOF disease (*n* = 1). Baricitinib was used exclusively in patients with JAK1 GOF disease. Abrocitinib was used only in patients with Netherton syndrome. Tofacitinib was prescribed for CARD14 deficiency and JAK1 GOF mutation, while upadacitinib was used in patients with Netherton syndrome and JAK1 GOF mutation. The most common indications for treatment were atopic dermatitis or eczematous dermatitis (*n* = 19, 90.5%), food allergy (*n* = 8, 38.1%), and asthma (*n* = 7, 33.3%). Most patients (*n* = 12, 57.1%) had multiple atopic manifestations.

The median (IQR) age at treatment initiation was 14 (9–16) years, ranging from 2 to 28 years. Post-treatment follow-up duration was reported for 19 patients, with a median (IQR) of 24 (8–24) months and a maximum of 6 years.

Treatment outcomes were reported for all 21 patients. A complete or major clinical response was described in 16 of 21 published patients (76.2%), a partial response in 4 (19.0%), and no reported response in 1 patient (4.8%) with Netherton syndrome. As above, these are descriptive frequencies among published patients and are not estimates of treatment effectiveness. Four patients (19.0%) were able to discontinue corticosteroid therapy. Previous or concurrent use of another targeted agent was reported in 9 patients (42.9%), most commonly another JAK inhibitor or dupilumab. In these patients the assessed response occurred during previous or concurrent exposure to another targeted agent and cannot be attributed to the JAK inhibitor alone.

Additional clinical benefits were reported in 15 patients (71.4%), most commonly increased appetite and resolution of food intolerance, and normalization of hepatic enzymes or reduction in hepatic inflammation. Paired pre- and post-treatment measurements were available for IgE in 5 patients and for eosinophils in 5; a reduction was recorded in 3 of 5 in each case. Adverse effects were reported in 5 patients (23.8%) and mainly included anaemia, lightheadedness with nausea, and body odour. Adverse-event assessment was explicitly documented in only a minority of reports. Given the small number of patients, the median follow-up, and the recognized under-reporting of adverse events in case reports, these data cannot exclude serious infection, cytopenia, thrombosis, or malignancy, particularly in patients with pre-existing impairment of antiviral, antifungal, or antimycobacterial immunity.

## 4 Discussion

The use of biologic agents and JAK inhibitors has increased in recent years, particularly in settings with broader access to targeted therapies. In inborn errors of immunity, their adoption has been slower, reflecting the heterogeneity of the underlying molecular defects, the need for genotype-informed therapeutic selection, and concerns regarding infectious complications and possible interference with already abnormal immune pathways (102). In addition, the chronic immune dysregulation in IEIs may induce epigenetic modifications that generates a vicious cycle of immune dysfunction and atopy, further influencing the treatment outcome (103). In this review, we examined the use of biologics and JAK inhibitors in inborn errors of immunity with atopic manifestations and described in detail dupilumab, omalizumab, and JAK inhibitors as the most frequently reported therapies.

Dupilumab was first approved in 2017 by the Food and Drug Administration (FDA) to be used for adult patients suffering from atopic dermatitis not completely controlled with conventional therapy. Later, it was extensively prescribed in a variety of atopic and autoimmune disorders, across a range of type 2 inflammatory conditions (98, 104, 105). In this review, dupilumab was primarily reported for the treatment of refractory atopy in STAT3 LOF mutation, Netherton syndrome, and DOCK8 deficiency and a complete or major clinical response was described in 82.2% of published patients with available outcome information. Additional reported clinical benefits across other disease domains were described in 68.3% of patients. Dupilumab binds a single target, IL-4R*α*, and thereby blocks signaling through both the type I and type II receptors for IL-4 and IL-13; the breadth of reported benefit reflects pleiotropic downstream effects of blocking this shared receptor subunit rather than action on multiple targets. The adverse effects were subtle, mostly conjunctivitis and a transient increase in eosinophil levels, which was consistent with a previous systematic review and meta-analysis study describing conjunctivitis in 26.1% of 3303 patients with atopic dermatitis (106). No biologic or JAK inhibitor holds a regulatory approval for atopic manifestations in IEIs, and all use described in this review is off-label with respect to the underlying disorder.

Omalizumab was first approved by the FDA in June 2003 for moderate-to-severe allergic asthma, and subsequent U.S. approvals expanded its use to chronic spontaneous urticaria (100) and, most recently, food allergy in February 2024. Labelled indications also include nasal polyps and reduction of allergic reactions after accidental food exposure (107). In this study, omalizumab was primarily prescribed for controlling atopic dermatitis and asthma in patients with STAT3 LOF mutation and DOCK8 deficiency. A complete or major clinical response was described in most published patients with STAT3 LOF disease. Notably, 37.5% of patients were able to discontinue corticosteroids, and several patients additionally benefited from a decrease in the infection rate, suggesting that omalizumab may be a useful option for patients with immunodeficiency who cannot tolerate corticosteroids or in whom they are contraindicated. These observations derive from a small number of uncontrolled published cases and require prospective confirmation.

The FDA first approved a JAK inhibitor in 2012, when tofacitinib was approved for rheumatoid arthritis; since then, the class has expanded to multiple indications across rheumatology, gastroenterology, dermatology, and hematology, such as rheumatoid arthritis, psoriatic arthritis, inflammatory bowel disease, atopic dermatitis, alopecia areata, and myelofibrosis. The indications for each isoform of JAK inhibitor across inborn errors of immunity (108) and other immune-mediated disorders (109) are reviewed elsewhere. In brief, ruxolitinib and baricitinib inhibit JAK1 and JAK2, tofacitinib acts predominantly on JAK1 and JAK3, and abrocitinib and upadacitinib are JAK1-selective. Their principal uses span myeloproliferative disease and graft-versus-host disease (ruxolitinib), rheumatological disease and alopecia areata (baricitinib and tofacitinib), and moderate-to-severe atopic dermatitis (abrocitinib and upadacitinib), with reported use in interferonopathies and in gain-of-function disorders of the JAK-STAT pathway. In this study, the most common agents used for the atopic manifestations in IEIs were ruxolitinib and baricitinib. A complete or major clinical response was described in 76.2% of published patients, and no major adverse reaction was reported in the available cases. No infection-related complications were reported in the small number of published patients treated with abrocitinib or upadacitinib. Given the very few patients and the short follow-up, this cannot be interpreted as evidence of a favorable infection profile in IEIs. In these patients, the ever-present concern for infectious adverse events, which are mostly viral, fungal, or mycobacterial and affecting respiratory and urinary tracts, as well as coagulopathy and malignant transformation, persists (87, 110). Also, infection-related safety signals have been described for these agents in other populations (111), which demands further investigations in IEIs.

JAK inhibitors are considered separately from monoclonal biologics here, because the two classes differ in mechanism, breadth of immunosuppression, adverse-event profile, monitoring requirements, and therapeutic intent. Four therapeutic models can be distinguished across the published experience. The first is pathway-corrective treatment, in which the agent acts directly on the causal signaling abnormality, as with ruxolitinib or baricitinib in JAK1, STAT5B, and STAT6 gain-of-function disease. The second is phenotype-directed treatment, in which a downstream atopic manifestation is targeted without correcting the underlying defect, as with dupilumab in STAT3 loss-of-function or in Netherton syndrome. The third is bridge therapy before HSCT, in which the objective is short-term disease control rather than durable remission. The fourth is symptomatic adjunctive therapy after failure of conventional treatment. These models differ in the outcomes that can reasonably be expected and should not be pooled into a single therapeutic conclusion.

Organized by mechanism rather than by frequency of use, the reported experience falls into seven groups: dominant type 2 signaling disorders, including STAT6 gain-of-function; JAK-STAT hyperactivation disorders, including JAK1 and STAT5B gain-of-function; combined immunodeficiencies with severe atopy, including DOCK8 deficiency and CARD11-related disease; hyper-IgE syndromes, including STAT3 loss-of-function and ZNF341 deficiency; epithelial-barrier disorders, including SPINK5-related Netherton syndrome; regulatory T-cell disorders, including IPEX; and other syndromic or incompletely classified conditions. Pretreatment screening and on-treatment monitoring for JAK inhibitors are summarized in Supplementary Table S6 based on a recent publication (112); these recommendations are extrapolated from non-IEI populations.

This study has several limitations. First, the inclusion criterion of an atopic phenotype may have led to overestimation, because in patients with inborn errors of immunity, manifestations resembling atopic disease may reflect underlying immune dysregulation rather than true IgE-mediated hypersensitivity. This concern is compounded by the fact that only a few studies performed standardized allergy testing. Second, the number of patients treated with each biologic was small, which limits the robustness of outcome interpretation; however, this is an expected consequence of the rarity of these conditions. Accordingly, we advocate for long-term longitudinal studies to better define clinical benefits, monitor safety, and characterize the immune profiles of patients receiving these agents. Such studies will not only strengthen the clinical application of precision medicine in inborn errors of immunity but may also uncover important mechanistic insights into the pathobiology of IEIs with atopic manifestations. Third, the frequencies reported here derive almost entirely from case reports and small series and are vulnerable to publication bias, selection bias, selective outcome reporting, non-publication of treatment failures, duplicate reporting, variable follow-up, inconsistent definitions of response, and confounding by concurrent therapies; they are descriptive summaries of published experience and not effectiveness estimates. Fourth, the certainty of the evidence is very low for every outcome assessed. Fifth, adverse events are systematically under-ascertained in reports whose primary purpose is to describe a successful treatment, and the median follow-up of 12 months is too short to detect malignancy, thrombosis, or late opportunistic infection; the maximum follow-up of six years was reached in very few patients. Sixth, a substantial proportion of patients received concomitant or prior systemic therapy, including corticosteroids, immunoglobulin replacement, antimicrobial prophylaxis, and other targeted agents, so improvement cannot be attributed to a single drug. Seventh, the review was not registered prospectively, which increases the risk of selective outcome reporting; a written protocol was agreed before screening and is available from the corresponding author.

## 5 Conclusion

Published case reports and small series suggest that biologics and JAK inhibitors may provide clinically meaningful control of selected atopic or atopic-like manifestations in some patients with inborn errors of immunity. Dupilumab has the largest published experience, while JAK inhibitors may be particularly relevant when the underlying disorder involves activation of the JAK-STAT pathway. However, the evidence is of very low certainty and is strongly affected by publication bias, heterogeneous outcome definitions, short follow-up, concurrent therapies, and incomplete safety reporting. Treatment should therefore remain individualized, genotype-informed, and accompanied by careful infection surveillance and longitudinal outcome assessment.

## Data Availability

The data analyzed in this study is subject to the following licenses/restrictions: The dataset is available upon request. Requests to access these datasets should be directed to m.jamee@garvan.org.au.
