# Supplementary material for: Biologics and JAK inhibitors for atopic manifestations in inborn errors of immunity: a systematic review
Source: Front Allergy. 2026 Oct 7;7:1867183. doi: 10.3389/falgy.2026.1867183 (PMC13683126; doi:10.3389/falgy.2026.1867183)
Supplement: Supplementary file 1 [file Table1.docx]

**Supplementary Table S1. Genotype-informed synthesis by mechanistic category**

| **Mechanistic category (representative genes)** | **Agents reported (n patients)** | **Biological rationale** | **Principal clinical outcome** | **Certainty of evidence** | **Infection risk / monitoring** | **HSCT still indicated?** |
| --- | --- | --- | --- | --- | --- | --- |
| Dominant type 2 signaling (STAT6 GOF) | Dupilumab (5); Ruxolitinib (1) | Germline GOF variants amplify IL-4/IL-13 signaling; IL-4Ra blockade removes the upstream driver of the constitutively active pathway, and JAK inhibition acts on the same axis. | n = 6. complete or major response 6/6. Reduction in eczema, food-triggered anaphylaxis frequency and respiratory infection burden; restrictive lung defect unchanged in the one patient in whom it was measured. | Very low | No signal of increased infection; fewer respiratory illnesses reported on dupilumab. Monitor for breakthrough anaphylaxis and angio-oedema. | Not established as an indication. Targeted blockade is first-line. |
| JAK-STAT hyperactivation (JAK1, STAT5B GOF) | Baricitinib (7); Ruxolitinib (4); Upadacitinib (4); Tofacitinib (1); Dupilumab (1) | GOF in JAK1 or STAT5B causes ligand-independent pathway activation. JAK inhibitors act proximally and are mechanistically matched; IL-4Ra blockade addresses only one downstream arm. | n = 17. complete or major response 12/17; partial 5. Improvement across dermatitis, eosinophilia and gastrointestinal inflammation; eosinophilia rebounded after ruxolitinib withdrawal in one patient. Response to a selective JAK1 inhibitor was consistently less complete than to JAK1/2 inhibition: three of the five partial responses are patients switched from or treated with upadacitinib rather than ruxolitinib or baricitinib. | Very low | Class-level risk of herpes zoster, viral reactivation and cytopenias. Monitor full blood count, lipids and liver enzymes; check VZV status and consider vaccination before starting. | Considered for refractory or multisystem disease; JAKi used for control or as a bridge. |
| Combined immunodeficiency with severe atopy (DOCK8, CARD11) | Dupilumab (37); Omalizumab (6) | Th2 skewing arises from defective cytoskeletal and TCR-signaling scaffolds. IL-4Ra blockade treats downstream type 2 inflammation and restores barrier and antimicrobial peptide function without correcting the underlying defect. | n = 43. complete or major response 33/43; partial 5; failure 2; not reported 3. Eczema and secondary skin infection burden improved. Dupilumab used pre-transplant as a bridge in DOCK8 deficiency. One paradoxical psoriasiform reaction reported. | Very low | Underlying susceptibility persists - viral (HSV, HPV, molluscum), bacterial and fungal. Continue prophylaxis and immunoglobulin replacement. Reported falls in bacterial skin infection reflect barrier improvement, not immune reconstitution. | Yes. HSCT remains the only curative option in DOCK8 deficiency and most CIDs; biologics give symptom control or a bridge and must not delay transplant. |
| Hyper-IgE syndromes (STAT3 LOF, ZNF341, IL6ST, IL6R, PGM3) | Dupilumab (45); Omalizumab (5); Benralizumab (1); Tralokinumab (1); multiple/sequential agents (1); Reslizumab, then benralizumab (1); Mepolizumab (1) | STAT3 dominant-negative disease raises IL-4Ra and IL-5 expression and impairs antimicrobial peptide production; type 2 blockade reduces eczema and restores barrier-associated defensin expression. IL-5/IL-5R and IgE blockade target the eosinophilic and ABPA phenotypes. | n = 55. complete or major response 50/55; partial 3; failure 1; not reported 1. Consistent reduction in topical corticosteroid and antibiotic requirement; serum IgE falls on dupilumab. Benefit in ABPA/mycoses and eosinophilic esophagitis reported but inconsistent. | Very low | No consistent increase; several reports describe fewer bacterial skin infections. Do not relax antimicrobial prophylaxis, chest surveillance or antifungal cover - lung disease remains the leading cause of death. | Selective. Performed in 6/164 in the NIH cohort with reduced infection burden but no effect on the skeletal and vascular phenotype. Biologics do not replace supportive care. |
| Epithelial-barrier disorders (SPINK5/Netherton syndrome) | Dupilumab (17); Secukinumab (7); Infliximab (3); Abrocitinib (2); Omalizumab (1); Upadacitinib (1); Ustekinumab (1) | LEKTI deficiency causes unopposed kallikrein activity, PAR2/NF-kB activation and TSLP release with Th2 skewing, on a background of IL-17/IL-36 signaling. Agents target either the type 2 arm (dupilumab) or the IL-17/IL-23 arm (secukinumab, ustekinumab). | n = 32. complete or major response 29/32; partial 2; failure 1. Eczema, pruritus and hair growth respond well to dupilumab; ichthyosis linearis circumflexa and scaling respond inconsistently. This dissociation is consistent with residual IL-17/IL-36 drive. | Very low | High baseline risk of staphylococcal sepsis in infancy. Normalization of the skin microbiome reported on dupilumab. Secukinumab and ustekinumab carry candidiasis risk. Continue microbiological surveillance. | No. Netherton syndrome is a keratinocyte-intrinsic barrier defect not corrected by hematopoietic replacement. |
| Regulatory T-cell disorders (FOXP3/IPEX, CTLA4, IL2RA) | Dupilumab (6) | Treg failure permits Th2 skewing of the Treg compartment and effector T-cell dysregulation. IL-4/IL-13 blockade relieves IL-4-mediated suppression of Treg number and CTLA-4 expression, with reported expansion of Treg and Th3 compartments. | n = 6. complete or major response 4/6; partial 1; failure 1. Cutaneous benefit is the most consistent finding. Enteropathy and endocrinopathy are not addressed by IL-4Ra blockade. | Very low | Background immunosuppression (sirolimus, corticosteroids) usually continues, and drives the infection risk. No dupilumab-attributable infections reported. | Yes. HSCT remains the only curative option for IPEX; dupilumab is adjunctive for refractory dermatitis. |
| Other syndromic or incompletely classified | Dupilumab (9); Omalizumab (3); Tofacitinib (1) | Heterogeneous. A shared Th2-skewed phenotype secondary to barrier failure or immune dysregulation rather than a single mechanism. | n = 13. complete or major response 10/13; partial 3. Improvement is largely dermatological. Magnitude is sometimes modest relative to the narrative claim (for example EASI 16.8 to 12.8 in hypohidrotic ectodermal dysplasia). | Very low | Disorder-specific. GATA2 and PIK3CD in particular retain substantial infection risk independent of biologic therapy. | Disorder-specific: indicated in GATA2 deficiency and some cases of APDS; not applicable in hypohidrotic ectodermal dysplasia or Rubinstein-Taybi syndrome. |

**Supplementary Table S2. Mapping of included disorders to the IUIS 2024 classification.**

| **Disorder as reported** | **Gene** | **Inheritance** | **IUIS 2024 table / category** | **Group (A/B/C)** | **Patients (n)** |
| --- | --- | --- | --- | --- | --- |
| STAT3 loss-of-function (dominant-negative) hyper-IgE syndrome | STAT3 | AD | Table 4 - Combined immunodeficiencies with associated or syndromic features | A | 52 |
| Netherton syndrome | SPINK5 | AR | Not IUIS-listed; syndromic epidermal differentiation disorder / primary atopic disorder | B | 32 |
| DOCK8 deficiency (AR hyper-IgE syndrome) | DOCK8 | AR | Table 4 - Combined immunodeficiencies with associated or syndromic features | A | 20 |
| JAK1 gain-of-function disease | JAK1 | AD | Table 6 - Diseases of immune dysregulation | A | 15 |
| CARD11 dominant-negative disease (CADINS) | CARD11 | AD | Table 4 - Combined immunodeficiencies with associated or syndromic features | A | 13 |
| STAT6 gain-of-function disease | STAT6 | AD | Table 6 - Diseases of immune dysregulation (added in the 2024 update) | A | 6 |
| IPEX syndrome | FOXP3 | XL | Table 6 - Diseases of immune dysregulation | A | 4 |
| Wiskott-Aldrich syndrome and WAS-related disorder | WAS | XL | Table 4 - Combined immunodeficiencies with associated or syndromic features | A | 4 |
| ZNF341 deficiency (AR hyper-IgE syndrome) | ZNF341 | AR | Table 4 - Combined immunodeficiencies with associated or syndromic features | A | 3 |
| Hyper-IgE phenotype, molecular diagnosis not established | Not identified; one heterozygous DOCK8 and one heterozygous PGM3 variant of uncertain significance | Not established | Cannot be mapped - causal variant not established | C | 3 |
| ARPC1B deficiency | ARPC1B | AR | Table 4 - Combined immunodeficiencies with associated or syndromic features | A | 2 |
| CTLA-4 insufficiency | CTLA4 | AD | Table 6 - Diseases of immune dysregulation | A | 2 |
| STAT5B gain-of-function disease (somatic, non-clonal) | STAT5B | Somatic | Table 6 - Diseases of immune dysregulation | A | 2 |
| Hypohidrotic ectodermal dysplasia | EDA | XL | Not IUIS-listed; ectodermal dysplasia with atopic-like dermatitis | C | 1 |
| Rubinstein-Taybi syndrome | CREBBP | AD | Not IUIS-listed; syndromic immunodeficiency reported but not a defined entity | C | 1 |
| Adenosine deaminase deficiency | ADA | AR | Table 1 - Immunodeficiencies affecting cellular and humoral immunity | A | 1 |
| Prolidase deficiency | PEPD | AR | Not a core IUIS IEI table; metabolic disorder with immune features | C | 1 |
| Loeys-Dietz syndrome with immune dysregulation | TGFBR2 | AD | Not IUIS-listed; connective tissue disorder with atopy | C | 1 |
| STK4 (MST1) deficiency | STK4 | AR | Table 1 - Immunodeficiencies affecting cellular and humoral immunity | A | 1 |
| 22q11.2 deletion (DiGeorge) syndrome | 22q11.2 deletion | De novo / AD | Table 4 - Combined immunodeficiencies with associated or syndromic features | A | 1 |
| PLCG2-associated disorder (PLAID / APLAID) | PLCG2 | AD | Table 7 - Autoinflammatory disorders | A | 1 |
| CARD14-mediated psoriasis (CAMPS) | CARD14 | AD | Table 7 - Autoinflammatory disorders | A | 1 |
| MALT1 deficiency | MALT1 | AR | Table 1 - Immunodeficiencies affecting cellular and humoral immunity | A | 1 |
| X-linked agammaglobulinaemia | BTK | XL | Table 3 - Predominantly antibody deficiencies | A | 1 |
| Activated PI3K-delta syndrome (APDS) | PIK3CD | AD | Table 1 - Immunodeficiencies affecting cellular and humoral immunity | A | 1 |
| CARMIL2 (RLTPR) deficiency | CARMIL2 | AR | Table 4 - Combined immunodeficiencies with associated or syndromic features | A | 1 |
| GATA2 deficiency | GATA2 | AD | Table 5 - Congenital defects of phagocyte number or function | A | 1 |

Group A = IUIS-listed inborn error of immunity (133 patients); Group B = primary atopic disorder or epithelial-barrier defect (32); Group C = syndromic or incompletely characterised condition (7). Total 172 patients. IUIS table numbers refer to the 2024 update of the IUIS phenotypic classification.

**Supplementary Table S3. Prespecified and post hoc sensitivity analyses.**

| **Analysis** | **Population included** | **Studies (n)** | **Patients (n)** | **Complete or major response, n / N (%)** | **Difference from the main analysis** |
| --- | --- | --- | --- | --- | --- |
| Main analysis | All included patients | 78 | 172 | 138/168 (82.1%) | Reference |
| Genetically confirmed IUIS-listed IEIs only | Group A of Supplementary Table S2 | 53 | 133 | 103/129 (79.8%) | Prespecified. Excludes epithelial-barrier disorders and conditions without an established molecular diagnosis. The response estimate falls by about two percentage points; direction and interpretation are unchanged. |
| Excluding epithelial-barrier disorders | Groups A and C only | 58 | 140 | 109/136 (80.1%) | Prespecified. Excludes Netherton syndrome only. Direction and magnitude unchanged. |
| Monotherapy only | No other new systemic intervention during assessment | Not estimable | Not estimable | Not estimable | Prespecified but not estimable. The source reports rarely give enough temporal detail to define an assessment window during which no other new systemic therapy was started. Reported as a limitation. |
| Patients for whom scoring system before and after treatment available | Validated instrument reported | 56 | 128 | 108/128 (84.4%) | Post hoc, added during revision. Restricted to patients in whom a severity instrument was recorded as assessed. Estimates from this subgroup are biased towards reports that scored their patients. |
| Follow-up at least 12 months | Patients with follow-up duration reported as 12 months or more | 40 | 91 | 75/90 (83.3%) | Prespecified. Follow-up duration is not reported for a substantial proportion of patients, so this analysis is restricted to those with a numeric duration. |

Complete or major response denominators exclude patients for whom the response was not reported or was not assessable. Freeman 2026 is excluded from every count because its dupilumab-treated patients overlap with James 2022 (Supplementary Table S4, P-001) and are reported in aggregate only. Pietzsch 2022 and Del Bel 2017 remain included publications but contribute no unique patient after merging, so 78 of the 81 included publications contribute at least one patient.

.

**Supplementary Table S4. Audit of overlapping publications and duplicate patients.**

| **Patient identifier** | **Publications reporting this patient** | **Matching variables used** | **Retained source** | **Reason for retention** | **Certainty of match (certain / probable)** |
| --- | --- | --- | --- | --- | --- |
| P-001 | James 2022 (13 NIH-enrolled patients); Freeman 2026 (28 dupilumab-treated patients) | Enrolling protocol NCT00006150; single centre (NIH Clinical Center); STAT3 genotype; dupilumab exposure; shared senior/corresponding author | James 2022 | Only source reporting individual patient data. Freeman 2026 reports the dupilumab-treated subgroup in aggregate and was not entered into the extraction sheet. | Certain at study level; probable at individual-patient level |
| P-002 | Submuth 2021; Murase 2021; Chu 2025 (Table 1 literature review) | Age, sex, Netherton phenotype (NS-ILC / NS-SE), dupilumab regimen and duration as tabulated by Chu | Submuth 2021; Murase 2021 | Chu 2025 contributes its own index case only; its Table 1 is a literature review of previously published patients, not new cases. | Certain |
| P-003 | Ollech 2021; Trombello 2025 (literature review, report R13) | Publication of origin, age at transplantation, anti-inflammatory pre-treatment | Ollech 2021 | Trombello 2025 contributes its own index case only; its review tabulates 53 previously published DOCK8 transplant recipients. | Certain |
| P-004 | Diaz-Cabrera 2021; Gualdi 2023; Dixit 2021; Staudacher 2021; Moraczewski 2021; Dick 2025a (Table 1 literature review) | Genotype, age, sex, dupilumab regimen and reported severity score | Primary reports | Dick 2025a contributes its three index cases only; its Table 1 is a literature review. | Certain |
| P-005 | Dick 2025a (Patient 1); Dick 2025b (Case 1) | Same centre (University of Minnesota), STAT3 dominant-negative disease, male sex | Both retained as distinct patients | Ages differ (8 years vs 15 years) and Dick 2025b reports the genotype (c.1909G>A, p.V637M) while Dick 2025a does not; judged to be different individuals. | Judged distinct - not a duplicate |
| P-006 | Sharma 2023 (16 patients with STAT6 GOF, 3 dupilumab-treated); Zangari 2024 (Pt 3, STAT6 GOF) | STAT6 gain-of-function genotype; shared co-authors and treating centre (Bambino Gesu Children’s Hospital, Rome) between the two publications; ethnicity, sex, age and which patients had actually started dupilumab | Both retained as distinct records | Resolved against the full texts. Sharma 2023 states that only P1, P2 and P6 had started dupilumab, and assigns each to a kindred of stated ethnicity: P1 to the Middle Eastern kindred A, P2 to the Hispanic kindred B (de novo p.D419A, female), and P6 to the East Asian kindred E (Hong Kong, p.D419G, a child with short stature and delayed bone age who gained height and weight on treatment). Zangari 2024 Pt 3 is a 23-year-old Caucasian Italian woman, so she cannot be any of the three Sharma records that were extracted, and the two sources do not double-count a patient. She is nonetheless likely to be Sharma 2023 P5: Sharma includes exactly one Italian kindred, led at Bambino Gesu by the senior author shared with Zangari 2024, and P5 is the patient on whom that group’s Olink proteomics platform was used. P5 had not started dupilumab when Sharma 2023 was submitted in October 2022 and was therefore never entered in the extraction sheet, so this residual patient-level overlap does not affect any count. | Judged distinct at record level – not a duplicate |
| P-007 | Steuer 2020; Andreasen 2020; Submuth 2021 (2 patients); Aktas 2020; Murase 2021 (2 patients); Inaba 2022 (Table 1 literature review) | Sex, age, treatment duration, response category and adverse effects as tabulated by Inaba | Primary reports | Inaba 2022 contributes its own index case only; its Table 1 is a literature review of the seven previously published Netherton syndrome patients treated with dupilumab, not new cases. Submuth 2021 and Murase 2021 are already captured under P-002; the Inaba table is the third publication to re-tabulate them. | Certain |
| P-008 | Sogkas 2020; Levy 2020; Dixit 2021; Lu 2021; Su 2021; Nihal 2022; Matucci-Cerinic 2022; Wang 2022; Staudacher 2022; James 2022; Shamriz 2022 (Table 3 literature review) | STAT3 variant, age at dupilumab initiation, sex, dupilumab regimen and baseline SCORAD as tabulated by Shamriz | Primary reports | Shamriz 2022 contributes its own two index patients (P1 and P2) only; its Table 3 is a literature review of 22 previously published dupilumab-treated patients with DN-STAT3 variants. Those 22 patients were attributed to their primary sources and were not entered separately from Shamriz 2022. | Certain |
| P-009 | Charvet 2021; Guillemin 2025 (Patient 19) | Treating network (Paris, with shared co-authors), CARD11 dominant-negative disease, age 47 years at dupilumab initiation, auricular polychondritis as the extra-cutaneous manifestation, adult regimen (600 mg on day 0 then 300 mg every 2 weeks) | Charvet 2021 (merged record) | Resolved by merger. Charvet 2021 supplies the variant (c.215G>A, p.Arg72Gln), the presenting features, the treatment history and SCORAD, a pruritus visual analogue scale and DLQI before and after treatment; Guillemin 2025 Patient 19 supplies the longer follow-up (28 months rather than 12) and a partial rather than complete response at that later time point. Both are now held in the single Charvet 2021 record and the Guillemin 2025 row has been deleted from the extraction sheet. | Probable |
| P-010 | Pietzsch 2022 (III.1); Diaz-Cabrera 2024 (Patient 4) | CARD11 c.223C>T (p.Arg75Trp), male sex, Dresden center and shared authors (Pietzsch, Schuetz), and identical SCORAD values before and on dupilumab (67 to 16.9) | Diaz-Cabrera 2024 (merged record) | Resolved by merger. Diaz-Cabrera 2024 supplies the dupilumab dose and interval (200 mg every 2 weeks), the age at onset and at diagnosis, the immunoglobulin E and eosinophil course and the 24-month outcome; Pietzsch 2022 III.1 supplies the numeric SCORAD (67 before dupilumab to 16.9 on treatment) and the German treating center, which have been carried across. Both are now held in the single Diaz-Cabrera 2024 record and the Pietzsch 2022 row has been deleted from the extraction sheet. | Certain |
| P-011 | Levy 2020; Guillemin 2025 (Patient 20) | ZNF341 autosomal recessive hyper-IgE syndrome (one patient in each report), French treating centres with shared co-authors, adult dupilumab regimen (600 mg on day 0 then 300 mg every 2 weeks) | To be decided - both provisionally retained | ZNF341 deficiency is exceptionally rare and the author of the 2020 report is a co-author of Guillemin 2025, but the recorded age at dupilumab initiation differs (48 years versus approximately 28 years), which argues for two different individuals. Confirm with the corresponding authors before submission. | Possible - not yet resolved |
| P-012 | Del Bel 2017 (II-2, III-1, III-2); Biggs 2022 (II-2, III-1, III-2) | Identical JAK1 c.1901C>A (p.A634D) variant, identical pedigree identifiers, same treating centre (BC Children’s Hospital, Vancouver), shared senior author (Turvey), identical ruxolitinib initiation ages (6 years and 20 months) and the same growth charts reproduced in both reports | Biggs 2022 (merged record) | Certain. Biggs 2022 cites Del Bel 2017 as its reference 2 and explicitly reports the follow-up of the same family over 6 years. The extraction sheet currently holds three Del Bel 2017 rows and two Biggs 2022 rows for what are three patients, so this pair is double-counted. Del Bel 2017 supplies the initial ruxolitinib dose (50 mg/m2/dose twice daily), the presenting phenotype and the failure of prednisone; Biggs 2022 supplies the 6-year follow-up, the maintenance dose after down-titration (1.5-2.5 mg/kg/day), the growth and hematological trajectories, the normalization of skin-prick testing and total immunoglobulin E, and the mother’s upadacitinib course. Resolved by merger. The three patients are now held as three Biggs 2022 records and the two redundant Del Bel 2017 rows have been deleted. The mother’s record has also been corrected from ruxolitinib, which she never received, to upadacitinib 15 mg once daily, and her response from complete to partial, which is what Biggs 2022 describes. | Certain |

*Entries P-002 to P-004, P-007 and P-008 record duplication risk arising from literature reviews embedded within primary reports rather than true duplicate publication. In each case only the index patients of the reviewing article were extracted and the reviewed patients were attributed to their primary source. P-009, P-010 and P-012 were confirmed duplicates and have been merged, so the extraction dataset holds 172 rather than 177 patients. P-011 remains unresolved and both records are provisionally retained.*

**Supplementary Table S5a. Risk-of-bias appraisal of case reports (Joanna Briggs Institute checklist, 8 items).**

| **Study (first author / year)** | **1** | **2** | **3** | **4** | **5** | **6** | **7** | **8** | **Overall judgement** |
| --- | --- | --- | --- | --- | --- | --- | --- | --- | --- |
| J. Yang, 2026 | Y | Y | Y | Y | Y | Y | Y | Y | Low risk of bias |
| C. Zhu, 2025 | Y | Y | Y | Y | Y | Y | Y | Y | Low risk of bias |
| Y. Vollmuth, 2025 | Y | Y | Y | Y | Y | Y | Y | Y | Low risk of bias |
| E. Uchiyama, 2025 | Y | Y | Y | Y | Y | Y | U | Y | Low to moderate risk of bias |
| S. Trombello, 2025 | Y | Y | Y | Y | Y | Y | Y | Y | Low risk of bias |
| S. Samra, 2025 | Y | Y | Y | Y | Y | Y | U | Y | Low to moderate risk of bias |
| A. Nanda, 2025 | Y | Y | Y | Y | Y | Y | U | Y | Low to moderate risk of bias |
| R. Johar, 2025 | Y | Y | Y | Y | Y | Y | U | Y | Moderate risk of bias |
| A. K. Y. Chu, 2025 | Y | Y | Y | Y | Y | Y | U | Y | Moderate risk of bias |
| T. Guo, 2025 | Y | Y | Y | Y | Y | Y | U | Y | Low to moderate risk of bias |
| B. E. Wilson, 2025 | Y | Y | Y | Y | Y | Y | Y | Y | Low risk of bias |
| C. Sun, 2025 | Y | Y | Y | U | Y | Y | Y | Y | Low to moderate risk of bias |
| J. M. Camino-Salvador, 2026 | Y | Y | Y | Y | Y | Y | Y | Y | Low risk of bias |
| P. Chokevittaya, 2024 | Y | Y | Y | Y | U | Y | Y | Y | Low to moderate risk of bias |
| W. Li, 2024 | Y | Y | Y | Y | Y | Y | Y | Y | Low risk of bias |
| G. Gualdi, 2024 | Y | Y | Y | Y | Y | Y | Y | Y | Low risk of bias |
| F. Alzahrani, 2024 | Y | Y | Y | Y | Y | Y | U | Y | Low to moderate risk of bias |
| P. A. Gutierrez-Marin, 2024 | Y | Y | Y | Y | U | U | U | Y | Moderate to high risk of bias for the therapeutic question |
| F. Liu, 2024 | Y | Y | Y | Y | Y | U | U | Y | Moderate risk of bias |
| R. A. Johar, 2023 | Y | Y | Y | U | Y | Y | U | Y | Moderate risk of bias |
| S. Gkalpakiotis, 2023 | Y | Y | Y | Y | Y | Y | Y | Y | Low risk of bias |
| L. K. Arruda, 2023 | Y | Y | Y | Y | Y | Y | Y | Y | Low risk of bias |
| A. S. Kao, 2023 | Y | Y | Y | Y | Y | Y | Y | Y | Low risk of bias |
| C. Caruso, 2023 | Y | Y | Y | Y | Y | Y | Y | Y | Low risk of bias |
| B. E. Wilson, 2023 | Y | Y | Y | Y | Y | Y | U | Y | Low to moderate risk of bias |
| S. Baris, 2023 | Y | Y | Y | Y | Y | Y | Y | Y | Low risk of bias |
| C. Zheng, 2023 | Y | Y | Y | Y | Y | Y | Y | Y | Low risk of bias |
| H.-J. Wang, 2022 | Y | Y | Y | Y | Y | Y | Y | Y | Low risk of bias |
| A. Nihal, 2022 | Y | Y | Y | Y | Y | Y | Y | Y | Low risk of bias |
| C. Matucci-Cerinic, 2022 | Y | Y | Y | Y | Y | Y | Y | Y | Low risk of bias |
| Y. Inaba, 2022 | Y | Y | Y | Y | Y | Y | Y | Y | Low risk of bias |
| K. H. Nguyen, 2022 | Y | Y | Y | Y | Y | Y | Y | Y | Low risk of bias |
| J. Lan, 2022 | Y | Y | Y | Y | Y | Y | Y | Y | Low risk of bias |
| P. Gupta, 2022 | Y | Y | Y | U | Y | Y | U | Y | Moderate risk of bias |
| C.-W. Lu, 2021 | Y | Y | Y | Y | Y | Y | Y | Y | Low risk of bias |
| M. C. Maher, 2021 | Y | Y | Y | U | Y | Y | U | Y | Moderate risk of bias |
| C. Dixit, 2021 | Y | Y | Y | Y | Y | Y | U | Y | Low to moderate risk of bias |
| E. Charvet, 2021 | Y | Y | Y | Y | Y | Y | Y | Y | Low risk of bias |
| T. P. Joshi, 2021 | Y | Y | Y | Y | Y | Y | Y | Y | Low risk of bias |
| A. Adatia, 2021 | Y | Y | Y | Y | Y | Y | U | Y | Low to moderate risk of bias |
| F. Cicek, 2021 | Y | Y | Y | Y | Y | Y | Y | Y | Low risk of bias |
| T. H. Andreasen, 2020 | Y | Y | Y | Y | Y | Y | U | Y | Low to moderate risk of bias |
| R. Levy, 2020 | Y | Y | Y | Y | Y | Y | Y | Y | Low risk of bias |
| G. Sogkas, 2020 | Y | Y | Y | Y | Y | Y | Y | Y | Low risk of bias |
| B. Steuer, 2020 | Y | Y | Y | U | Y | Y | U | Y | Moderate risk of bias |
| M. Jesenak, 2020 | Y | Y | Y | Y | Y | Y | Y | Y | Low risk of bias |
| D. V. Osypchuk, 2020 | Y | Y | Y | Y | Y | Y | U | Y | Low to moderate risk of bias |
| C. N. Gruber, 2020 | Y | Y | Y | Y | Y | Y | Y | Y | Low risk of bias |
| K. Blanchard, 2020 | Y | Y | Y | U | Y | Y | U | Y | Moderate risk of bias |
| S. Volc, 2020 | Y | Y | Y | Y | U | Y | U | Y | Moderate risk of bias |
| A. D. Yalcin, 2015 | Y | Y | Y | U | U | Y | U | Y | Moderate risk of bias |

Y = yes, N = no, U = unclear, NA = not applicable. Items: 1, demographic characteristics clearly described; 2, patient history clearly described and presented as a timeline; 3, current clinical condition on presentation clearly described; 4, diagnostic tests or assessment methods and results clearly described; 5, intervention or treatment procedure clearly described; 6, post-intervention clinical condition clearly described; 7, adverse events or unanticipated events identified and described; 8, takeaway lessons provided. Item 4 was rated unclear where the diagnostic work-up was named but its results were not reported, item 5 where the agent was named but the dose and interval were not stated, and item 7 where the report made no explicit statement about tolerability.

**Supplementary Table S5b. Risk-of-bias appraisal of case series (Joanna Briggs Institute checklist, 10 items) and cohort studies.**

| **Study (first author / year)** | **Design** | **1** | **2** | **3** | **4** | **5** | **6** | **7** | **8** | **9** | **10** | **Overall** |
| --- | --- | --- | --- | --- | --- | --- | --- | --- | --- | --- | --- | --- |
| S. Blunder, 2025 | Case series (prospective pilot, n = 3) | Y | Y | Y | U | U | Y | Y | Y | Y | Y | Moderate risk of bias |
| J. K. Dick, 2025a (Pediatr Dermatol) | Case series (n = 3) | U | U | Y | U | U | Y | Y | Y | U | NA | Moderate risk of bias |
| J. K. Dick, 2025b (JAAD Case Rep) | Case series (n = 3; 1 eligible) | U | N | Y | U | U | Y | Y | Y | U | NA | Moderate to high risk of bias |
| A. E. James, 2022 | Case series (n = 17) | Y | N | Y | U | U | Y | Y | Y | U | NA | Moderate risk of bias |
| A. F. Freeman, 2026 | Prospective cohort (n = 164; 28 dupilumab-treated) | Y | N | Y | Y | U | Y | Y | U | Y | Y | Moderate risk of bias for the therapeutic question |
| N. Alsediq, 2025 | Retrospective observational cohort with prospective follow-up (n = 10; 9 treated for atopic dermatitis) | Y | Y | Y | Y | Y | Y | Y | Y | Y | Y | Low risk of bias |
| C. Guillemin, 2025 | Multicentre retrospective study (n = 37; 30 extracted, 29 retained after de-duplication) | Y | U | Y | U | U | Y | Y | Y | Y | Y | Low to moderate risk of bias |
| H. Li, 2025 | Case series (n = 2) | N | N | Y | N | U | Y | Y | Y | Y | NA | Moderate risk of bias |
| S. Fujita, 2025 | Case series (n = 2; both upadacitinib-treated, both extracted) | N | Y | Y | U | Y | U | Y | Y | Y | NA | Low to moderate risk of bias |
| E. Liquidano-Perez, 2024 | Retrospective case series (n = 11; 2 dupilumab-treated) | Y | N | Y | Y | Y | Y | Y | U | Y | Y | Moderate to high risk of bias for the therapeutic question |
| P. Zangari, 2024 | Case series (n = 6; 5 on dupilumab at report) | U | Y | U | U | U | Y | Y | Y | Y | NA | Moderate risk of bias |
| N. M. Diaz-Cabrera, 2024 | Case series (n = 6; 5 dupilumab, 1 omalizumab) | U | Y | Y | N | U | Y | Y | Y | Y | NA | Moderate risk of bias |
| M. E. Horesh, 2024 | Genetic association study (n = 59 variant carriers; 1 baricitinib-treated) | N | Y | Y | N | N | U | Y | U | U | NA | Moderate to high risk of bias for the therapeutic question |
| M. Sharma, 2023 | International case series (n = 16; 3 dupilumab-treated) | N | U | Y | N | U | Y | Y | U | U | NA | Moderate risk of bias for the therapeutic question |
| A. Fayand, 2023 | Case series (family, n = 9; 7 JAKi -treated: 6 baricitinib, 1 upadacitinib) | Y | N | Y | Y | Y | Y | Y | Y | Y | NA | Low to moderate risk of bias |
| N. S. Salici, 2023 | Case series (n = 2 siblings) | N | N | Y | U | Y | Y | Y | Y | Y | NA | Moderate risk of bias |
| S. Yan, 2022 | Prospective case series (n = 4) | Y | Y | Y | U | U | Y | Y | Y | Y | Y | Low to moderate risk of bias |
| O. Staudacher, 2022 | Case series (n = 3) | U | Y | Y | U | U | Y | Y | Y | Y | NA | Moderate risk of bias |
| O. Shamriz, 2022 | Retrospective case series (n = 3; 2 dupilumab-treated) | Y | Y | Y | U | Y | Y | Y | Y | Y | NA | Low to moderate risk of bias |
| L. Pietzsch, 2022 | Family study / case series (n = 6 affected; 1 dupilumab-treated, merged into Diaz-Cabrera 2024) | U | N | Y | U | U | Y | Y | U | Y | NA | Moderate to high risk of bias for the therapeutic question |
| C. M. Biggs, 2022 | Case series (family, n = 3; 2 ruxolitinib- and 1 upadacitinib-treated; retained source for P-012) | U | N | Y | U | Y | Y | Y | Y | Y | NA | Moderate risk of bias |
| C. Gan, 2022 | Case series (n = 2) | N | N | U | U | Y | Y | Y | Y | Y | NA | Moderate risk of bias |
| K. Submuth, 2021 | Case series (n = 2) | U | Y | U | U | U | Y | Y | Y | Y | NA | Moderate risk of bias |
| C. Murase, 2021 | Case series (n = 2) | U | Y | Y | U | U | Y | Y | Y | Y | U | Moderate risk of bias |
| A. Ollech, 2021 | Retrospective case series (n = 9; 2 dupilumab-treated) | Y | Y | Y | Y | Y | Y | Y | U | Y | NA | Low to moderate risk of bias |
| R. Eisenberg, 2021 | Case series (n = 2) | U | N | Y | U | U | Y | Y | Y | Y | NA | Moderate risk of bias |
| N. Gomes, 2020 | Case series (n = 2) | N | U | Y | U | U | Y | Y | Y | Y | NA | Moderate risk of bias |
| T. Novice, 2020 | Case series (n = 3; 1 omalizumab-treated) | N | N | Y | U | U | Y | Y | N | Y | NA | Moderate to high risk of bias for the therapeutic question |
| I. Luchsinger, 2020 | Case series (compassionate use, n = 4) | Y | Y | Y | U | U | Y | Y | Y | Y | Y | Low to moderate risk of bias |
| K. L. Del Bel, 2017 | Case series (family, n = 3; 2 ruxolitinib-treated, merged into Biggs 2022) | N | N | Y | U | Y | Y | Y | Y | Y | NA | Moderate risk of bias |

*Items (JBI case series): 1, clear inclusion criteria; 2, condition measured in a standard and reliable way; 3, valid methods for identification of the condition; 4, consecutive inclusion; 5, complete inclusion; 6, demographics reported; 7, clinical information reported; 8, outcomes or follow-up clearly reported; 9, presenting site demographics reported; 10, statistical analysis appropriate. Item 2 was rated N where no validated severity instrument was applied before and after treatment. Cohort studies were appraised with the Joanna Briggs Institute checklist for cohort studies. Liquidano-Perez 2024, Sharma 2023 and Horesh 2024 are appraised for the therapeutic question only, being primarily descriptive genotype-phenotype studies in which biologic treatment is incidental.*

**Supplementary Table S6. Pre-treatment screening and on-treatment monitoring investigations for patients with inborn errors of immunity starting a JAK inhibitor**

| **Test** | **Comments** |
| --- | --- |
| **Laboratory** | |
| Complete blood count | - Initially then every 1-2 weeks over the first 3 months then every 10 weeks or as clinically indicated |
| Renal and liver function tests | - Initially then every 1-2 weeks over the first 3 months then every 10 weeks or as clinically indicated |
| Lipid profile | - Initially then every 3 months unless indicated |
| Coagulation profile | - Initially then periodically as per patient’s condition |
| Inflammatory markers (ESR, CRP) | - Initially then periodically as per patient’s condition |
| Infectious organisms (PCR based where appropriate) | - Hep B and C, HSV, VZV, EBV, CMV, HIV, and tuberculosis initially. Monitor for tuberculosis, EBV, CMV, HSV yearly or as per-patient’s condition |
| Lymphocyte subsets (CD3, CD4, CD8, CD19, CD16/56) | - Initially |
| IgG, A, and M serum levels | - Initially |
| **Vaccination** | |
| Vaccination status | - Initially. Update vaccination status. Live vaccines (if not otherwise contraindicated) should be given > 4 weeks before starting JAKi and with-held afterwards - Give seasonal influenza vaccine and recombinant zoster vaccine as scheduled |
| Antiviral, antifungal, antimicrobial prophylaxis | - Should be tailored according to patient’s underlying disease, type and rate of infectious complications, structural lung damage and with considering drug side effects and interaction with JAKi |
|  |  |
| **Imaging** | |
| Chest X-ray | - Initially. |
| Abdominal US, Low dose Chest CT (in patients with pulmonary involvement) and Brain MRI (including vasculitis protocol) | - Initially and every 2 to 3 years or as clinically indicated |
| ECG and Cardiac Echo | - Initially then ECG yearly as indicated |
| **Dose reduction** | - Non disease related cytopenia: Hemoglobin < 6.5 g/dL, platelets < 25,000/ul, neutrophils < 500/ul) or worsening disease related cytopenia - Elevated liver enzymes ALT, AST, and ALP > 2.5X upper limit of normal (ULN) or total bilirubin level > 2.5X ULN unless liver involvement is disease related. - Renal insufficiency Creatinine clearance < 30 mL/min; dose adjustment to 50% of previous dose. - Comedications Strong CYP3A4 inhibitors (ie, posaconazole) or dual CYP2C9/CYP3A4 inhibitors (ie, fluconazole); consider dose adjustment to 50% of previous dose. - Infections Chronic or recurrent viral infections such as HPV, HSV, CMV, VZV, viral hepatitis. |
| **Discontinuation** | - Pregnant or breast-feeding. - Acute VTE not related to underlying disease. - Cardiovascular events. - Malignancy not in remission - Drug hypersensitivity. - Severe active infections, acute or chronic, including latent tuberculosis and opportunistic infections. - Severe organ dysfunction if affecting drug metabolism (e.g., GFR < 30 mL/min/1.73 m2 or liver enzymes 5X ULN, or more than grade II toxicity). |
| **Peri-transplantation** | - JAKi should be tapered during conditioning and discontinued before stem cell infusion, - After engraftment, temporary reintroduction of JAKi or alternative immunosuppression may be necessary if withdrawal symptoms occur |
